# A Case of Primary Malignant Mixed Mesonephric Tumor of the Vagina

**DOI:** 10.7759/cureus.88468

**Published:** 2025-07-21

**Authors:** Shinichiro Maeda, Michiyasu Miki, Takuya Aoki, Shinya Yoshioka, Shigeo Hara

**Affiliations:** 1 Department of Obstetrics and Gynecology, Kobe City Medical Center General Hospital, Kobe, JPN; 2 Department of Obstetrics and Gynecology, Kyoto Okamoto Memorial Hospital, Kyoto, JPN; 3 Department of Obstetrics and Gynecology, Okubo Hospital, Akashi, JPN; 4 Department of Pathology, Kobe City Medical Center General Hospital, Kobe, JPN

**Keywords:** adenocarcinoma of the vagina, concurrent chemoradiotherapy, fertility preserving, histopathologic diagnosis, immunohistochemistry, laparoscopic surgery, malignant mixed mesonephric tumor, mesonephric adenocarcinoma, rare tumors, vaginal carcinosarcoma

## Abstract

Mesonephric adenocarcinoma is a clinically aggressive, rare malignant neoplasm characterized by mesonephric (Wolffian) differentiation. It can occur throughout the female genital tract; however, most cases arise in the uterine cervix. Vaginal mesonephric adenocarcinoma is uncommon, and cases with a concomitant sarcomatous component are extremely rare. Using appropriate immunohistochemistry, we present a rare case of a primary malignant mixed mesonephric tumor of the vagina, defined by biphasic epithelial and mesenchymal malignant components of mesonephric origin. The patient was a 34-year-old woman who presented with vaginal pain and bleeding. Pelvic examination and diagnostic imaging revealed a primary vaginal tumor measuring approximately 4 cm without distant metastases. Histological examination of the biopsy specimen revealed an adenocarcinoma with a sarcomatous component. Our initial diagnosis was primary vaginal carcinosarcoma; however, additional immunohistochemistry of the tumor revealed luminal membranous positivity for CD10, diffuse nuclear staining for TTF1, and estrogen receptor negativity. These findings led to a final diagnosis of a primary malignant mixed mesonephric tumor of the vagina. Because of the patient's young age and desire for fertility preservation, curative concurrent chemoradiation was performed after oocyte and ovarian cryopreservation and migration via laparoscopic surgery. The tumor showed a marked reduction in size within three months and remained under control. This case emphasizes the need for clinicians and pathologists to consider the possibility of a mesonephric origin when encountering vaginal adenocarcinoma with a sarcomatous component. Appropriate immunohistochemical analysis is essential for establishing an accurate diagnosis and developing effective therapeutic strategies.

## Introduction

Mesonephric adenocarcinoma (MA) of the female genital tract is a rare tumor originating from the mesonephric duct remnants. MAs typically occur along the original trajectory of mesonephric (Wolffian) ducts, most commonly in the lateral wall of the uterine cervix [1,2], and some cases are accompanied by a sarcomatous component [3]. MA is uncommon in the vagina, and vaginal MA with a sarcomatous component is extremely rare, with only two cases reported in the literature [2,4,5]. The marked rarity of this tumor likely reflects, at least in part, the diagnostic challenges posed by its under-recognition and morphological overlap with other adenocarcinomas. Accurate diagnosis, however, is essential for guiding appropriate therapeutic strategies. Here, we present a case of vaginal adenocarcinoma with a sarcomatous component, which was confirmed by appropriate immunohistochemistry to be a rare case of a primary malignant mixed mesonephric tumor of the vagina.

## Case presentation

Clinical summary

A 34-year-old Japanese woman (gravidity 1 and parturition 0) presented with vaginal pain and intermittent vaginal bleeding for two months at a local gynecological clinic. She was referred to our hospital with a mass on the left vaginal wall. Cytological smear obtained from the vaginal tumor and stained using the Papanicolaou method indicated adenocarcinoma. At our hospital, a pelvic examination revealed an irregularly shaped, crater-like hard mass on the left wall of the lower vagina, approximately 2-3 cm from the vaginal orifice, without cervical or vulvar involvement. The mass bled easily on palpation. A transvaginal ultrasound examination revealed an irregular tumor sized 41 × 36 × 30 mm. Her uterus, bilateral adnexa, and parametria were normal on pelvic examination and transvaginal ultrasound screening.

The patient was healthy and had no medical history other than the use of oral contraceptives. She had no history of radiation, hormones, or intrauterine diethylstilbestrol exposure. She had no allergies and never smoked. The patient’s family history included colorectal cancer in her grandfather, both colorectal and prostate cancer in her paternal great-uncle, and ovarian cancer in her mother’s cousin. Blood tests showed that the serum levels of general gynecological tumor markers (cancer antigen 125, cancer antigen 19-9, and carcinoembryonic antigen) were within the normal range. The squamous cell carcinoma antigen levels were slightly elevated (1.3 ng/mL) compared to the institutional normal range (0.0-1.2 ng/mL).

**Figure 1:**
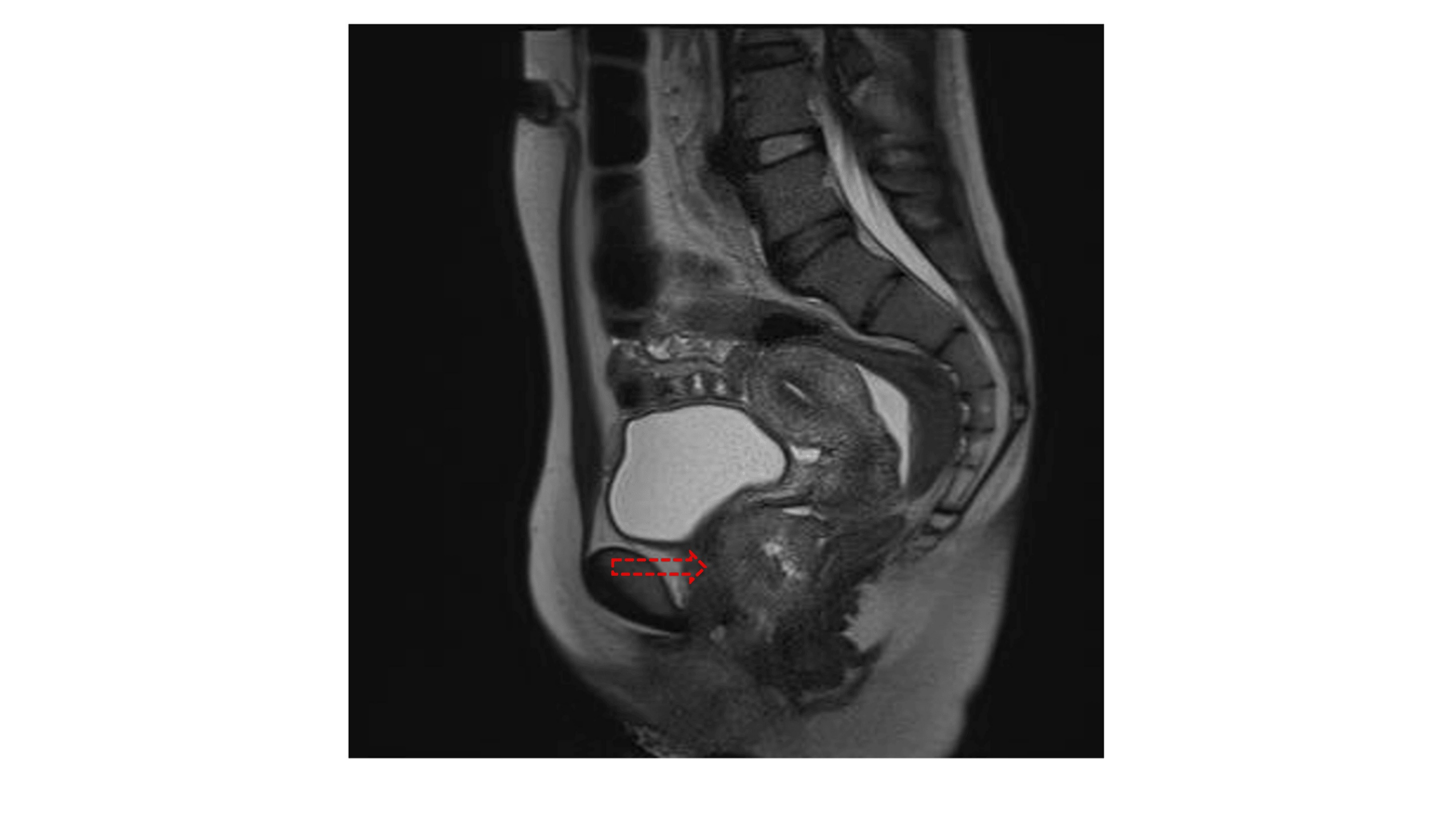
T2-weighted MR imaging of the pelvis in a sagittal section (red arrow, irregular mass on the vagina)

Magnetic resonance imaging revealed an irregular mass on her vagina growing outward into the vaginal space (Figure 1).

Computed tomography showed no distant metastases, including the pelvic lymph nodes. Positron emission tomography revealed fluorodeoxyglucose accumulation in the vaginal tumor but showed no signs of distant metastases.

Histological examination of the biopsy specimen revealed predominant proliferation of atypical glands and mitotic spindle cells, indicating adenocarcinoma with a sarcomatous component. The initial diagnosis was primary vaginal carcinosarcoma (stage 1, T1bN0M0). Given the patient's young age and strong desire for fertility preservation, curative concurrent chemoradiation was selected for treatment, based on the general therapeutic strategies for vaginal cancer [6,7]. Given the size and location of the tumor, primary surgical treatment would have required pelvic exenteration with hysterectomy, iliac lymph node dissection, and possibly combined resection of the bladder, urethra, and rectum. Such an approach would have resulted in permanent loss of fertility and a significant impact on her quality of life, particularly in terms of sexual and urinary function. Instead, concurrent radiotherapy and chemotherapy were successfully administered after oocyte and ovarian cryopreservation and laparoscopic surgery for ovarian migration to mitigate the effect of pelvic irradiation on the ovarian reserve. The tumor showed a marked reduction in size within three months and remained under control.

Pathological findings

**Figure 2:**
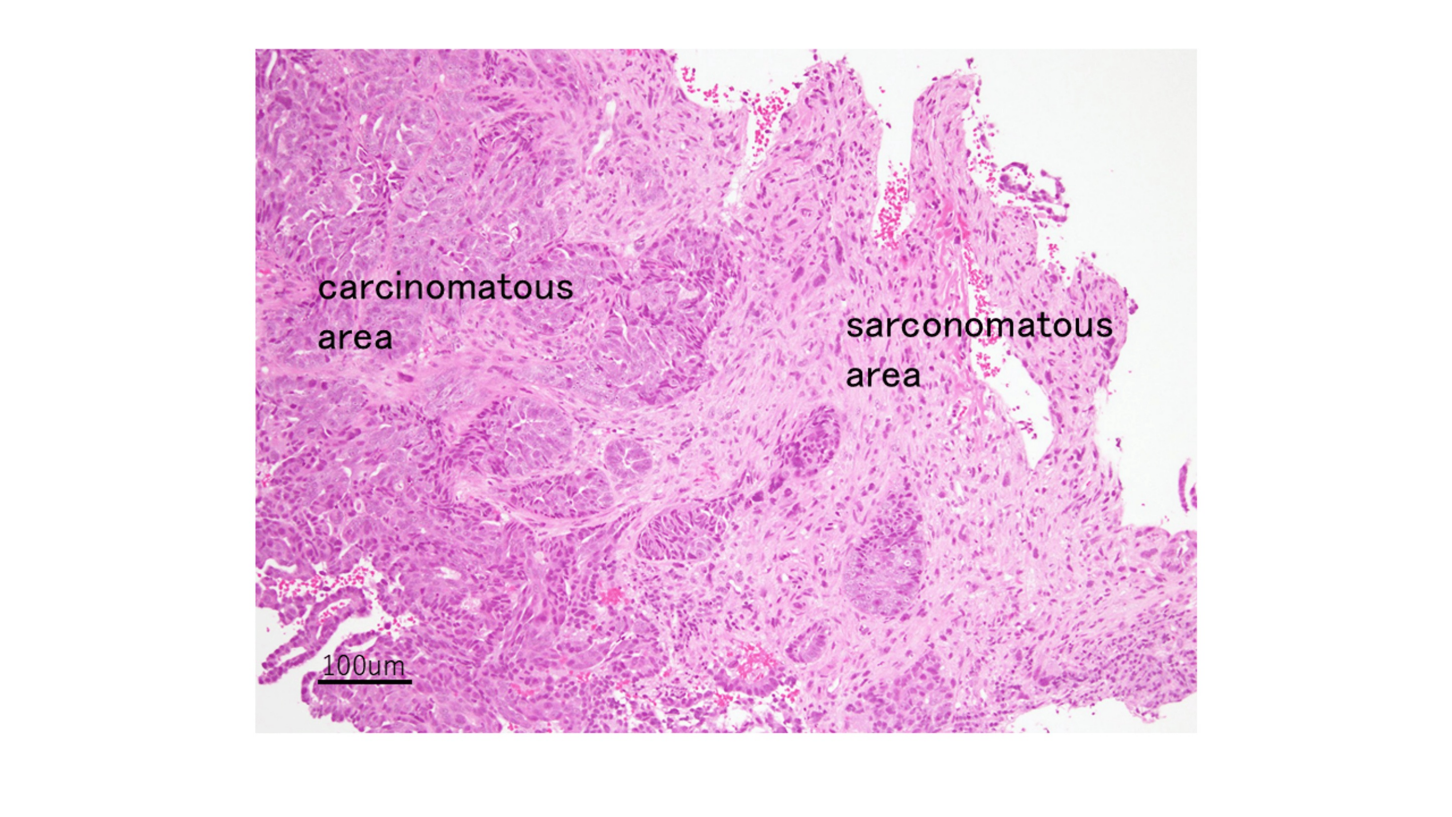
Carcinomatous and sarcomatous tumor cells, mostly demarcated, are merged with gradual transition (Hematoxylin and Eosin stain, 10× magnification)

Microscopic examination of the biopsy specimen revealed that the neoplasm had a biphasic malignant morphology with both epithelial and mesenchymal components. The two elements were mostly separated and demarcated but partially merged with a gradual transition (Figure 2).

The epithelial components included adenocarcinomas with tubular and ductal patterns, back-to-back glands, and malignant nuclear features with mitosis. The mesenchymal components were nonspecific, undifferentiated malignant spindle cells with mitoses.

**Figure 3:**
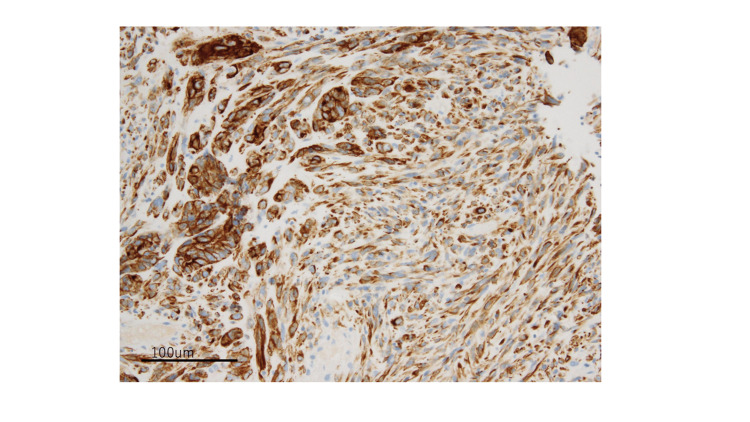
Positive CK AE1/AE3 stain of the tumor cells, both epithelial and mesenchymal (20× magnification) CK - cytokeratin

The initial immunohistochemical staining workup indicated that the epithelial and mesenchymal tumor cells were diffusely positive for AE1/AE3 and negative for p16 (Figure 3).

The results of further staining for immunohistochemical markers are shown in Table 1.

**Table 1:** Staining results for immunohistochemical markers + indicates positive staining; – indicates negative staining CK - cytokeratin; ER - estrogen receptor;

Marker	Epithelial component	Mesenchymal component
CK AE1/AE3	+	+
ER	-	-
GATA3	-	-
CD10	luminal membranous	-
Calretinin	-	-
Vimentin	-	+
TTF1	+	-
P53	- (null pattern)	- (null pattern)

**Figure 4:**
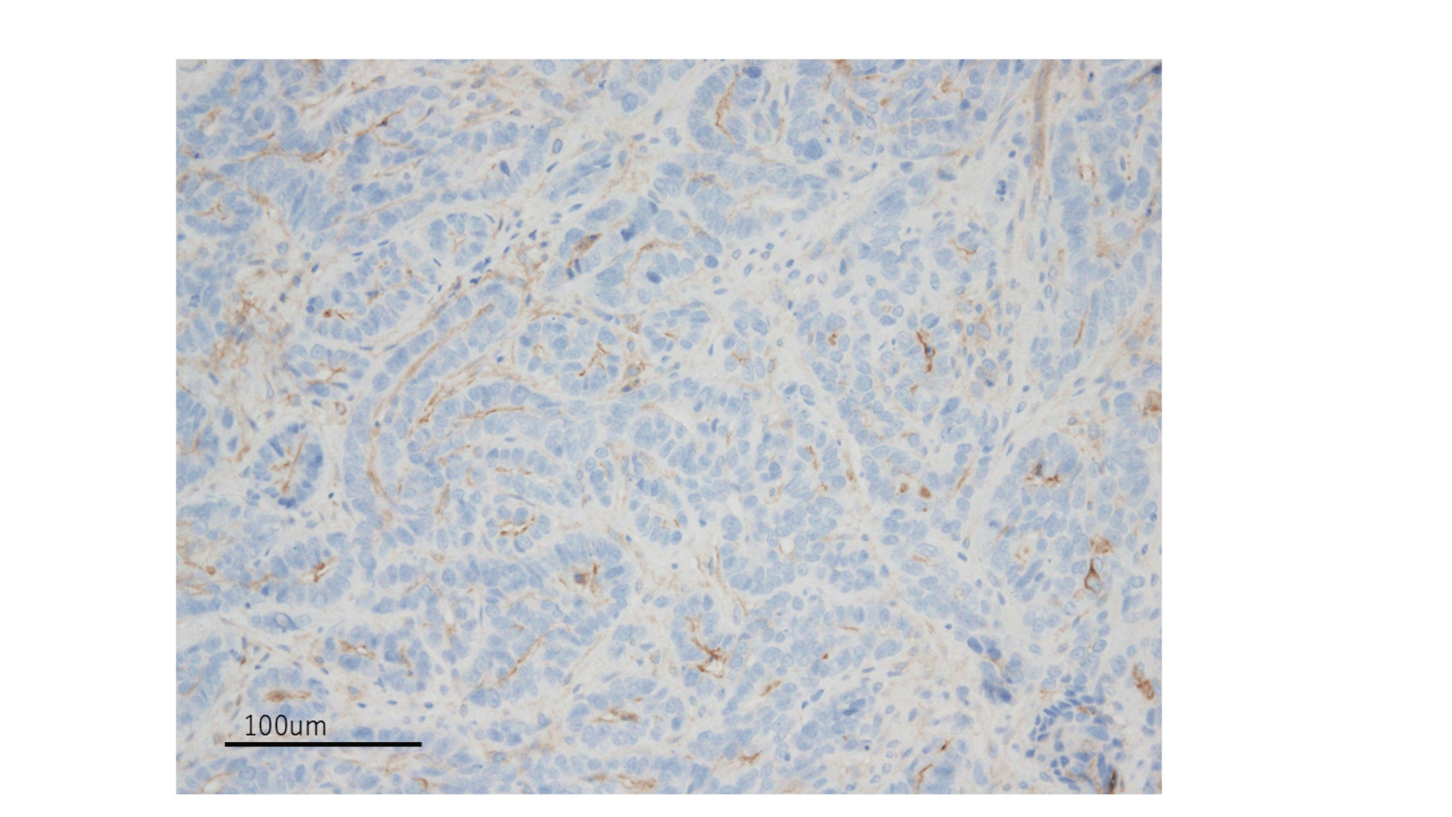
The epithelial glandular component showing luminal membranous positivity for CD10 (20× magnification)

**Figure 5:**
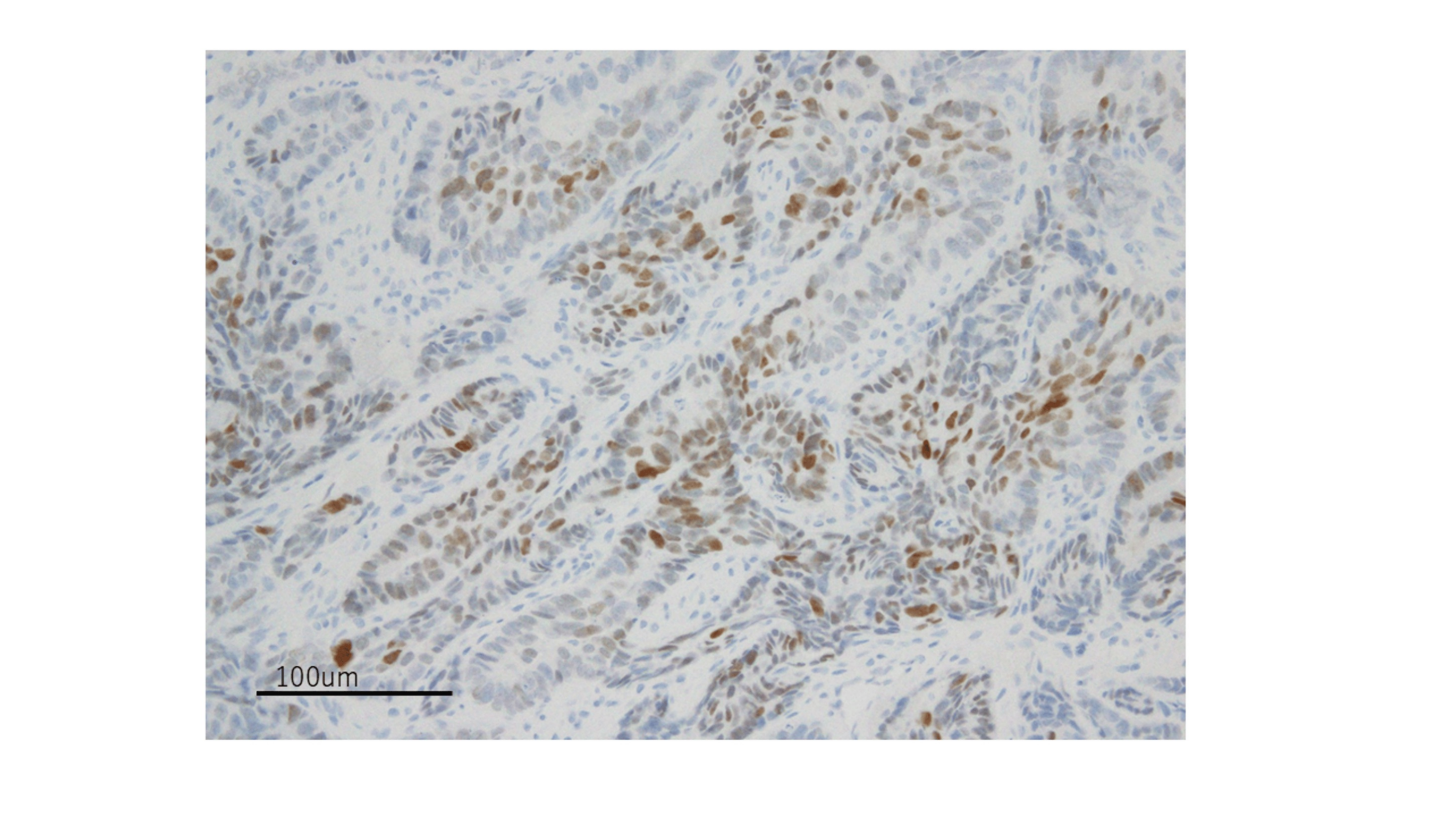
Diffuse nuclear staining for TTF1 in the epithelial tumors (20× magnification)

The carcinomatous glandular component showed luminal membranous positivity for CD10 and diffuse nuclear staining for TTF1 (Figures 4, 5).

**Figure 6:**
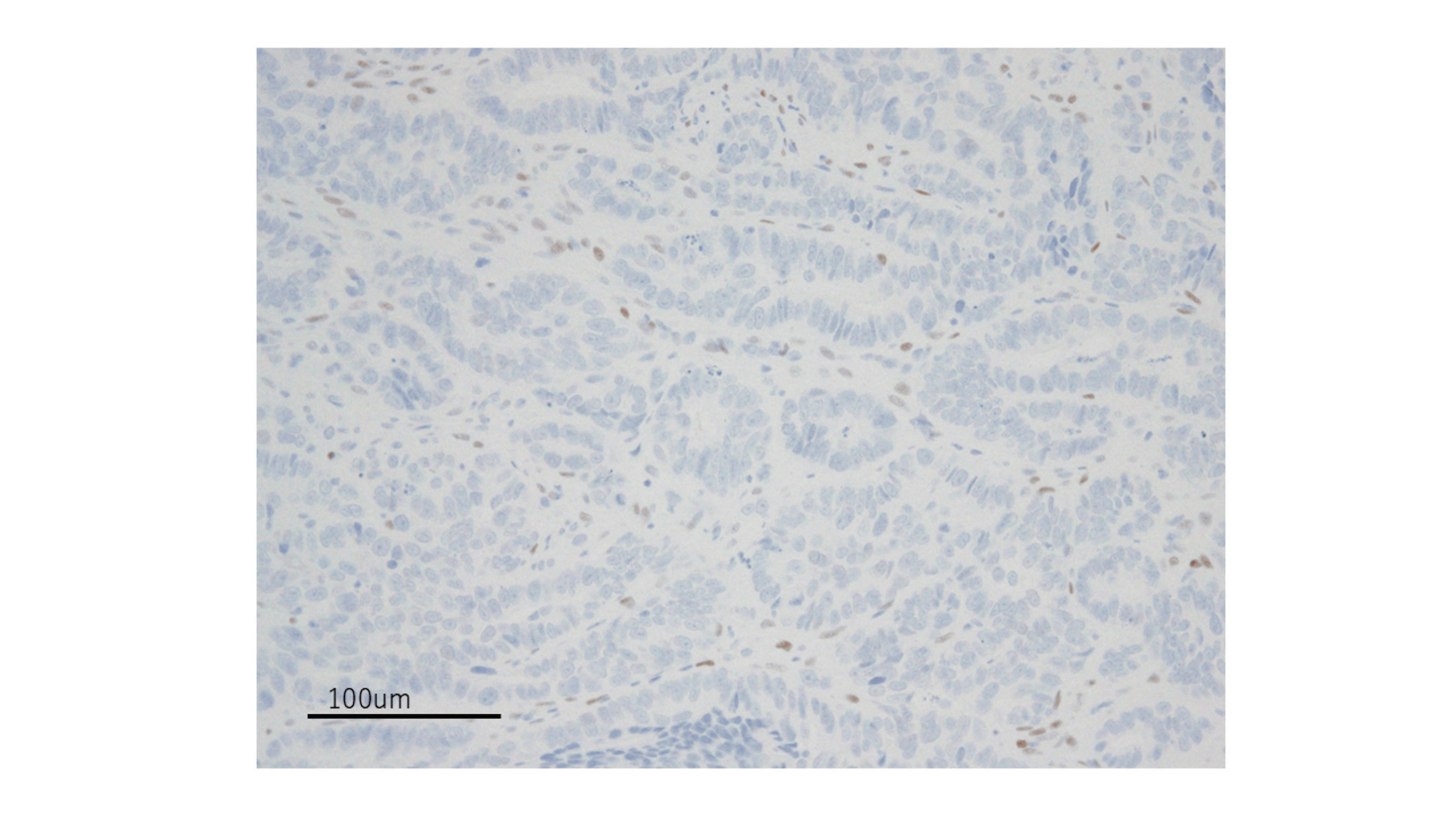
Negative ER stain of the tumor cells (20× magnification) ER - estrogen receptor

The sarcomatous component was positive for vimentin. Both epithelial and mesenchymal components were negative for the estrogen receptor (ER) (Figure 6).

**Figure 7:**
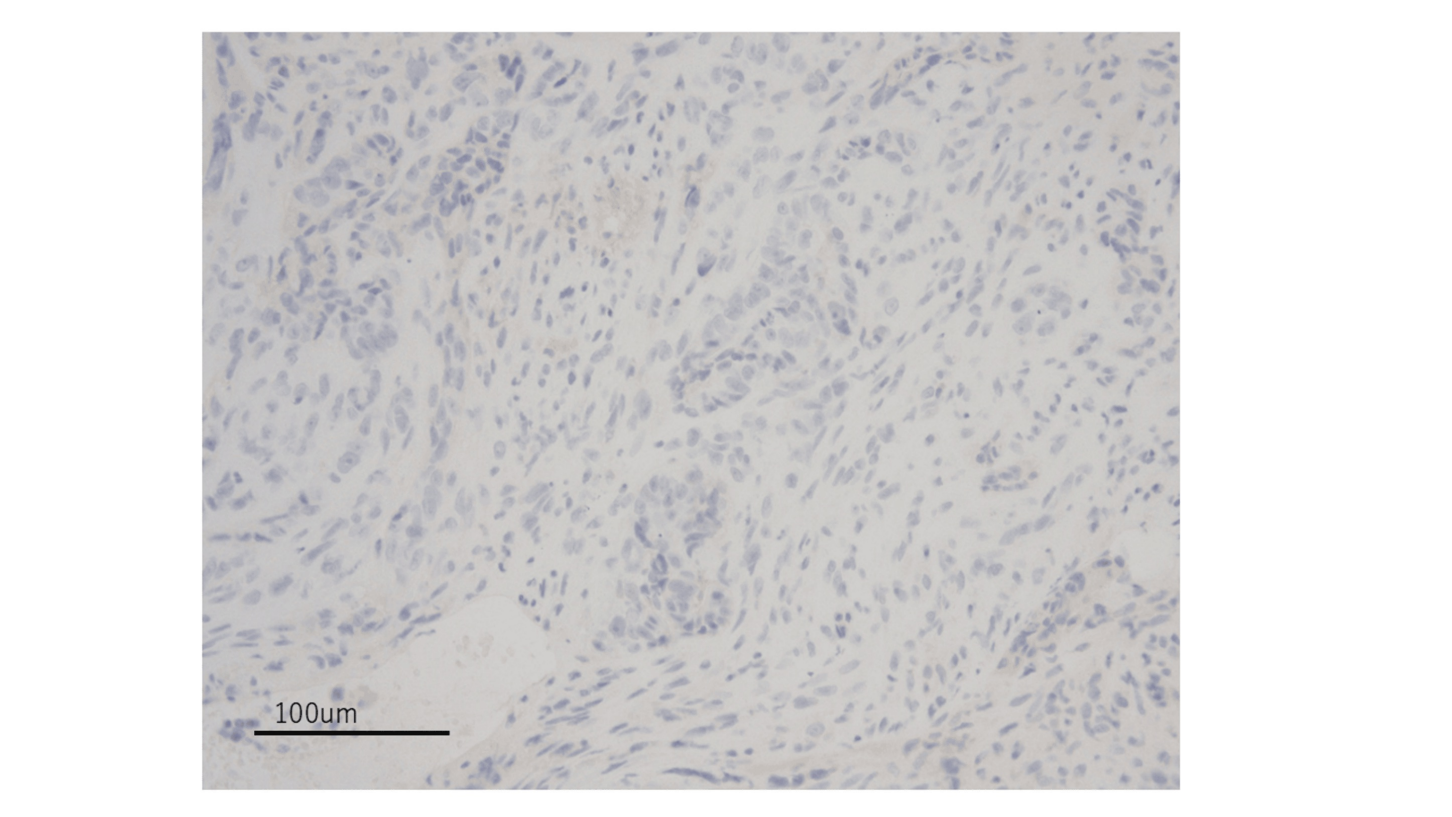
Complete loss of p53 expression of the tumor cells (20× magnification)

Based on these findings, a final diagnosis of a primary malignant mixed mesonephric tumor of the vagina was made. Additionally, both the epithelial and mesenchymal components demonstrated a null pattern of p53 staining (Figure 7), suggesting an underlying TP53 mutation.

## Discussion

MA can occur throughout the female genital tract; however, most arise in the uterine cervix [1,2,8]. When MA arises in the uterine corpus (endometrium) or ovary, it is often described as a mesonephric-like adenocarcinoma (MLA) [9]. MAs are uncommon in the vagina. Primary vaginal carcinoma accounts for only 1-2% of all gynecological malignancies, and vaginal MA represents <0.1% of vaginal cancers [10]. Ferrari et al. conducted a systematic review and listed 13 reported cases of vaginal MAs since 2004 [10]. Lee et al. compiled 23 cases of vaginal MAs dating back to 1954 [11].

The rarity of vaginal MA is partially attributed to the difficulty in diagnosis. Pathologically, MAs exhibit various architectural patterns, including tubular, ductal, solid, papillary, retiform, and sex-cord-like within the same tumor [12]. MAs can be easily misclassified as other differential diagnoses, including clear cell carcinoma, endometrial carcinoma, and other types of adenocarcinomas. Eosinophilic luminal material is a desirable presentation. Mesonephric remnants or background hyperplasia may help recognize MA [12]; however, these mesonephric features are not essential markers for diagnosing MAs, as malignant parts could have overgrown them, or there could be a sampling issue [13]. In our case, neither eosinophilic material nor mesonephric remnants were observed in the biopsy specimens. Therefore, MA poses a diagnostic challenge for clinicians and pathologists.

Recent studies, however, have identified valuable markers for distinguishing them from their morphological mimics. After conducting a large-scale study comparing the usefulness of GATA-3, TTF-1, CD10, and calretinin for identifying MAs and MLAs, Pors successfully defined immunohistochemical and molecular profiles of MAs and MLAs [2,9]. Thus, the current diagnostic criteria for MA or MLA are considered immunohistochemical positivity for at least one of GATA3, TTF1, CD10 (luminal), or calretinin, and negative/focal positivity for ER or, where applicable, molecular confirmation of KRAS (Kirsten rat sarcoma viral oncogene homolog) mutations [9,14]. In our case, immunohistochemical TTF1 positivity, luminal membranous CD10 staining, and ER negativity were the key features in determining the mesonephric origin. Molecular testing was not performed due to the absence of health insurance coverage.

A drawback of this diagnostic method is that sarcomatous components can accompany MAs and MLAs. For example, a previous study showed that 10 of 42 MAs were accompanied by sarcomatous components [3]. With biphasic features composed of epithelial and mesenchymal components, such tumors are often called malignant mixed mesonephric tumors or mesonephric carcinosarcomas.

In our case, the mesenchymal components lacked immunohistochemical features of mesonephric differentiation, such as TTF-1 expression or luminal CD10 staining. This loss of mesonephric marker expression in the sarcomatous component may be attributed to epithelial-mesenchymal transition (EMT), a phenomenon known to underlie sarcomatous differentiation in gynecologic carcinosarcomas [15]. Recent studies have shown that mesonephric markers such as GATA3 and CD10 may be lost in sarcomatous areas, supporting an EMT-related process [2, 5].

A study from a United States (US) database found as many as 49 cases of vaginal carcinosarcoma between 1988 and 2010 in a catchment area that represents 27.8% of the US population [16]. Therefore, it is natural to assume that vaginal carcinosarcomas are more numerous than previously reported by a series of case reports. However, the possibility of mesonephric origin has been examined in a few cases of vaginal carcinosarcoma. To our knowledge, only two cases of vaginal malignant mixed mesonephric tumor have been reported in the English literature [4,5].

Accurate diagnosis of mesonephric origin for vaginal carcinosarcomas is clinically important, as evidence continues to accumulate regarding the behavior of mesonephric tumors in other gynecologic sites. For example, emerging data suggest that they are associated with an unfavorable prognosis and a propensity for distant recurrence, particularly in the lungs [9]. Our concurrent chemoradiation therapy following laparoscopic surgery for fertility preservation has been successful, but optimal management strategies for mesonephric carcinomas and carcinosarcomas in particular may evolve in the future as more cases are reported.

Given the rarity of vaginal mesonephric tumors, much remains to be elucidated regarding their biological behavior and prognosis. For instance, complete loss of p53 expression in our case, indicating a TP53 mutation, is unusual and a matter for further research, as most MAs and MLAs have shown a wild-type staining pattern for p53 [11]. Whether the relatively poor prognosis of mesonephric tumors in other sites applies to vaginal cases remains unclear and requires further case accumulation. Given the limited understanding of the tumor, accurate identification and diagnosis of vaginal MA and malignant mixed mesonephric tumors are essential for characterizing their clinical behavior and developing appropriate therapeutic strategies.

## Conclusions

To the best of our knowledge, this is the third documented case of a primary malignant mixed mesonephric tumor of the vagina. Clinicians and pathologists should consider the possibility of a vaginal adenocarcinoma with a sarcomatous component of mesonephric origin. If a mesonephric origin is suspected, a diagnosis should be confirmed by immunohistochemical examination.
